# Job demands and functional resources moderating assistant and Registered Nurses' intention to leave

**DOI:** 10.1002/nop2.694

**Published:** 2020-11-20

**Authors:** Andrea Eriksson, Göran Jutengren, Lotta Dellve

**Affiliations:** ^1^ School of Engineering Sciences in Chemistry, Biotechnology and Health KTH Royal Institute of Technology Huddinge Sweden; ^2^ Department of Work Life and Social Welfare University of Borås Borås Sweden; ^3^ Faculty of Health and Welfare Østfold University College Halden Norway; ^4^ Department of Sociology and Work Science Gothenburg University Gothenburg Sweden

**Keywords:** assistant nurses, intention to leave, job demands‐resources model, registered Nurses

## Abstract

**Aims:**

To investigate how job demands and resources interact with each other to predict intention to leave among assistant nurses and Registered Nurses.

**Design:**

Longitudinal study.

**Methods:**

Questionnaire data were collected yearly during three years (October 2012–December 2014) from Registered Nurses (RN) and assistant nurses (*N* = 840) employed in Swedish hospitals. Associations and interaction effects of demands and resources were assessed with correlation analyses and regression models.

**Results:**

Job demands predicted assistant nurses' intentions to leave, while resources predicted RNs' intention to leave. For RNs, several resources were functional in moderating the associations between demands and intention to leave: social support, vertical trust, and humanity moderated work pace and workflow moderated emotional demands. For assistant nurses, organizational clarity and interprofessional collaboration moderated emotional demands. None of the resources had a moderating effect on the associations between quantitative demands or illegitimate tasks and intention to leave.

## 1 INTRODUCTION

The high turnover rates among nurses are one of the largest challenges for healthcare organizations to deliver service today. At the same time, organizational conditions forming demands and resources seem to play an important role in the high turnover. Work‐related reasons for nurses' intentions to leave include poor organization of work, working conditions and social work climate (Hasselhorn et al., 2005; Heinen et al., 2013). However, nurses who leave their current position do not necessarily leave their profession. A recent Swedish study by Rudman et al. (2019) shows that almost 90% of trained, Registered Nurses (RN) still work as nurses 10–15 years after getting their nursing licence. There are, however, indications that an increased proportion of nurses are leaving their positions in Swedish public hospitals for employments with other healthcare providers (Rudman et al., 2019).

Healthcare organizations, including hospitals, might thus need to improve their handling of today's unwanted high turnover among nurses. The job demands‐resources model (JD‐R‐model, Demerouti et al., 2001) has the potential to increase our understanding of how interactions between specific demands and resources contribute to staff turnover. To support the development of functional resources that better meet job demands for assistant nurses and RNs, exploratory analysis of interactions between specific job demands and resources was conducted in this study. Intention to leave in this study includes those nurses who are considering looking for another job, that is considering leaving their current workplace.

### 1.1 Background

The JD‐R‐model integrates stress research with research on motivational factors (Bakker & Demerouti, 2017; Demerouti & Bakker, 2011; Demerouti et al., 2001). According to the model, there are different resources in work buffering for demands in work. Resources include physical, social or organizational factors that are functional for fulfilling work goals, that stimulate learning and development and that reduce the physical and psychological costs of demands (Bakker et al., 2003). Examples of job demands are high work pressure or emotional demands connected to patient work. Functional and moderating job resources include role clarity or support from supervisors and colleagues (Bakker & Demerouti, 2007). The JD‐R‐model points to the importance of individuals having resources in their working environment to counteract negative effects of high job demands. The model builds on assumptions that a lack of job resources is hindering individual goal fulfilment that can contribute to feelings of failure and frustration, which in turn can contribute to distancing oneself from work and decreased work engagement (Bakker et al., 2003; Demerouti et al., 2001). These mechanisms are also related to the intention to leave (Hom et al., 2012).

**Figure 1:**
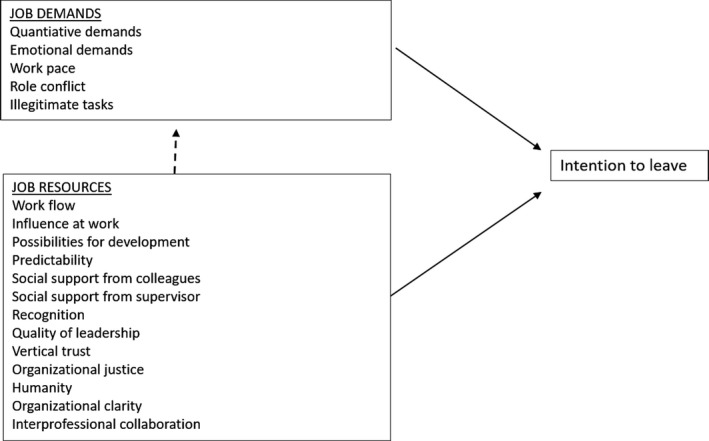
Job demands and job resources included in the study as potential demands and resources affecting intention to leave among assistant nurses and Registered Nurses

Demands and resources in work have been shown to be associated with work engagement, job satisfaction, burnout, sick leave and intention to leave in different working sectors (Bakker & Demerouti, 2007; Demerouti & Bakker, 2011; Tims et al., 2013). There are numerous studies showing the importance of health factors like job satisfaction and exhaustion for nurses' intentions to leave (Aiken et al., 2002; Havaei et al., 2016; Hayes et al., 2006; Ito et al., 2001; Perry et al., 2016; Sveinsdóttir & Blöndal, 2014). However, there is more limited research on how specific resources interact with specific job demands and how they might have a functional impact that buffers the importance of demands on nurses' intentions to leave. The JD‐R‐model highlights that demands differ between different jobs and that resources need to be functional to meet the specific demands that specific occupations face in work (Demerouti et al., 2001). A recent literature review has identified work overload, lack of formal rewards, work–life interference, supervisor support, authentic management, transformational leadership, interpersonal relations, autonomy and professional resources as key job demands and resources for nurses (Broetje et al., 2020). More specifically, previous research points to demands with significance for the intention to leave and staff turnover among nurses including quantitative and qualitative demands connected to patient work, role conflicts, time pressure and illegitimate tasks (Chan et al., 2013; Hasselhorn et al., 2005; Hayes et al., 2012). This study has thus included quantitative demands, work pace, role conflict and illegitimate tasks as job demands which potentially are associated with nurses' intention to leave (see Figure 1).

Resources at work that are associated with nurses' intentions to leave embrace general perceptions of resources in the work environment (Chan et al., 2013). Specific resources that have been identified affecting nurses intention to leave include influence at work, possibilities for development (Carter & Tourangeau, 2012; Hayes et al., 2012; Tummers et al., 2013), interprofessional collaboration, leadership practices (Choi et al., 2013), support from managers and colleagues (Hasselhorn et al., 2005; Hayes et al., 2012; Ito et al., 2001), recognition and predictability (Strömgren, 2017a). These resources have thus been included in this study as job resources which are potentially associated with nurses' intention to leave (see Figure 1). Overall, job resources related to social capital and an organizational culture of reciprocity have been shown to be associated with intention to leave and turnover of healthcare professionals, including nurses (Stordeur et al., 2007; Strömgren, 2017a). A study by Stordeur et al. (2007) has, for example, shown that hospitals with low staff turnover seem to be characterized by an organizational culture of trust and justice. Trust in the healthcare organization has also been identified as related to intention to leave in a study of hospital nurses (Bobbio & Manganelli, 2015). Overall, it has been argued that a work culture characterized by trust, fairness and respect promotes staff retention in health care (Wong & Cummings, 2009). Vertical trust, organizational justice and humanity (i.e. a workplace culture characterized by respect and reciprocity; Oxenstierna et al., 2008) have thus been included in this study as job resources which potentially are associated with nurses' intention to leave (see Figure 1). Finally, factors related to professional work and ward practices, including work processes, have also been shown to affect nurses' intention to leave (Choi et al., 2013). Organizational clarity (i.e. clarity of how work is organized including role clarity, Pejtersen et al., 2010) and workflow were thus also included in this study as job resources which potentially are associated with nurses' intention to leave.

In summary, there is extensive previous research on how job demands and resources are associated with affect nurses' intention to leave. However, there is more limited research analysing whether specific resources moderate the association between specific demands and nurses' intentions to leave. One previous cross‐sectional study analysed the interaction between workplace social capital on job demands and healthcare workers' intention to leave (Strömgren, 2017b). Another cross‐sectional study has investigated if perceptions of collective efficacy in workgroups in elderly care moderated the association between physical workload and intention to leave (Parry, 2008).

One previous study has applied the JD‐R‐model for investigating how different job demands and resources impact RN's intentions to leave (Moloney et al., 2018). There are, however, few studies focusing on assistant nurses in hospitals or comparing RNs' and assistant nurses' intentions to leave. Assistant nurses often share work situations with RNs but might have different job demands, work activities and roles at work. The classifications of assistant nurses and RNs vary between countries. In most countries including Sweden, RNs have an examination from a nursing programme at the university level and they receive a regulated licence. RNs might lead or supervise care delivered by assistant nurses who are responsible for the basic care of patients. In Sweden, an assistant nurse takes an examination in college, but the job title is not licensed. In Canada and the United States, persons carrying out similar work tasks in hospitals as Swedish assistant nurses might be labelled as licensed practical nurses. One cross‐sectional Canadian study compared RNs and licensed practical nurses. Havaei et al. (2016) demonstrated that intention to leave due to poor career advancement was more common among RNs compared with licensed practical nurses. Licensed practical nurses more frequently reported experience with physical job demands, inability to provide safe care, having too much responsibility and health problems and symptoms of burnout (Havaei et al., 2016). In Sweden, assistant nurses' labour market differs from that of RNs, which might be significant for their turnover opportunities (SCB, 2017).

### 1.2 Aims

The aims of this study were to investigate what job demands and resources predict the intention to leave and what resources moderate the associations between job demands and intention to leave among assistant nurses and RNs. The more specific job demands and resources investigated in this study are presented in Figure 1.

### 1.3 Design

Questionnaire data collected at three points in time (one year apart) from assistant nurses and RNs at five Swedish hospitals were analysed with regression models stratified by occupational group.

## 2 METHOD

### 2.1 Sample/Participants

Two small hospitals (approximately 100 beds and 700 employees) and three mid‐sized hospitals (approximately 500 beds and 4,000 employees) located in urban areas were selected for the study. Nurses employed at three to five care units in each of the five participating hospitals were included in the study. The care units were selected so that their areas of specialization would match across hospitals to the greatest extent possible. The selection from each hospital included an emergency unit, an acute medical and surgical intake unit (alternatively, at the small hospitals, an intensive care unit [ICU]) and one medical and one surgical ward. However, in one of the hospitals, the two units/wards from the surgical department declined to participate due to engagement in other ongoing research and development work. RNs and assistant nurses who had been working at least 6 months at the same unit were offered to participate in the study. At T3, the questionnaire was only offered to those who had participated in the study at T1 and/or T2. The response rate was 67% (*N = *621) at T1, 71% at T2 (*N = *607) and 73% at T3 (*N = *424). In total, 840 individuals, 516 RNs and 324 assistant nurses participated in the study.

### 2.2 Data collection and measures

Questionnaires were administered in 2012 (T1), 2013 (T2) and 2014 (T3) during the period October 2012 to December 2014. Depending on preferences of the included units, the questionnaire was distributed by email or in a sealed envelope. Non‐responders received up to two reminders.

The outcome measure *Intention to leave* was measured by a single question (“How often have you thought about applying for another job?”) from the second version of the Copenhagen Psychosocial Questionnaire (COPSOQ II; Pejtersen et al., 2010). The responses were rated on a 5‐point scale from 1 “no, to a very low degree”–5 “yes, to a very high degree” (i.e. high level of perceived intention to leave).

The included measures of explanatory variables represented job demands and resources (see Table 1). Most of the job demands and resources were measured by validated instruments from the COPSOQ II (Pejtersen et al., 2010). However, the resources *humanity* and *organizational clarity* and the job demand *illegitimate tasks* were measured with an instrument validated by Oxenstierna et al. (2008). Furthermore, the organizational resource *interprofessional collaboration* was measured using an instrument developed from a qualitative study by Lindgren et al. (2013). All items were rated on a 5‐point scale with 5 representing a high level of perceived intention to leave. All independent items were checked for multicollinearity.

**Table 1:** Overview of measures included in the study and the internal consistency of indexes

Measurements	Cronbach's alpha
Intention to leave (1 item)	–
JOB DEMANDS at T1	
Quantitative demands	0.80
Emotional demands (3 items)	0.67
Work pace (3 items)	0.84
Role conflict (3 items)	0.70
Illegitimate tasks (1 item)	–
JOB RESOURCES at T1	
Workflow (1 item)	–
Influence at work (3 items)	0.74
Possibilities for development (3 items)	0.74
Predictability (2 items)	0.61
Social support from colleagues (2 items)	0.60
Social support from supervisor (3 items)	0.84
Recognition (3 items)	0.79
Quality of leadership (8 items)	0.94
Vertical trust (1 item)	–
Organizational justice (4 items CA 0.76)	0.76
Humanity (3 items. CA 0.89)	0.89
Organizational clarity (4 items)	0.85
Interprofessional collaboration (4 items)	0.85

### 2.3 Validity and reliability

Most of the job demands and resources and intentions to leave were measured with instruments from COPSOQ II that have been tested and validated in countries worldwide. A validated Swedish translation of the instruments from COPSOQ II was used for this study (https://www.copsoq-network.org/validation-studies/). Complementary instruments used in this study were developed and validated in Swedish: *Illegitimate tasks* were measured with an instrument validated by Oxenstierna et al. (2008), the organizational resource *interprofessional collaboration* was measured using an instrument developed from a qualitative study by Lindgren et al. (2013). All independent items were checked for multicollinearity. Cronbach's alpha of the different instruments was acceptable ranging from 0.60–0.89 (see Table 1). The instruments in this study with only two items manifested the lowest Cronbach's alpha, which is common for instruments with fewer items (Vaske et al., 2017).

### 2.4 Ethics

The study was approved by the regional ethical research committee in Stockholm, Sweden. Informed consent was applied.

### 2.5 Data analysis

The analyses were performed in three steps. As a first step, explorative analyses were performed to give a descriptive overview of the data for the current sample. Therefore, analyses of mean values were performed for comparing assistant nurses and RNs in terms of intention to leave, job demands and resources. These comparisons were performed using a series of Student's *t* tests.

In the second step, Pearson correlation analyses, stratified into occupational groups, were performed for bivariate associations between variables of interest to investigate what kinds of demands and resources at T1 predicted intention to leave at T2 and T3. The correlation was classified as weak at *r* = 0.20 and below, fair at *r* = 0.21–0.40, moderate at *r* = 0.41–0.60, strong at *r* = 0.61–0.80 and very strong at *r* = 0.81–1.00 (Altman, 1990).

In the third step of the analyses, a series of (stepwise selection) were used to investigate whether a specific resource at T1 moderated the association between a specific demand at T1 and intention to leave at T2 and T3. This meant that it was analysed if each one of 13 different resources moderated the association between each one of 5 different demands and intention to leave at T2 and T3 for assistant nurses and RNs, generating a total of 260 regression models. In each model, first one of the specific job demands was added (e.g. quantitative demands), then one of the specific resources was added (e.g. quality of leadership) and finally the product of the specific job demands (e.g. quantitative demands) and the specific resource (e.g. quality of leadership) was added. Before creating the interaction term, the predictors (each specific demand and specific resource) were centred by subtracting each value from its respective mean to achieve a better estimate of the interaction term (Aiken et al., 1991). Statistical significance was considered when *p *< .05. JMP version 10.0.2 (SAS Institute, Cary, NC, USA) was used for all statistical calculations.

## 3 RESULTS

### 3.1 Descriptive and comparative statistics

Most participants were women and RNs. Less than half of the RNs,and the majority of the assistant nurses, had worked in their current occupation for more than 14 years (Tables 2 and 3). Compared with assistant nurses, RNs rated statistically higher mean values of intention to leave only at T3. In all job demands except emotional demands and illegitimate tasks, RNs rated statistically higher compared with assistant nurses (see Table 4). Assistant nurses rated predictability, organizational clarity and interprofessional collaboration higher compared with RNs. The only resource that RNs rated slightly higher was possibilities for development (see Table 4).

**Table 2:** Characteristics of the study group

	All		RNs		Assistant nurses	
	*N*	%	*N*	%	*N*	%
All	840	*100*	516	*61*	324	*39*
Sex						
Female	549	*90*	339	*89*	210	*91*
Male	62	*10*	42	*11*	20	*8*
Years in occupation						
< 2 years	26	*6*	21	*8*	5	*3*
2–7 years	75	*18*	61	*24*	14	*9*
8–14 years	94	*22*	68	*27*	26	*16*
>14 years	223	*53*	105	*41*	118	*72*

**Table 3:** Number of RNs and assistant nurses answering the survey at T1, T2 and T3

	All	RNs	***%***	Assistant nurses	***%***
	*N*	*N*		*N*	
T1	621	385	62	236	38
T2	607	365	60	242	40
T3	424	247	58	177	42

**Table 4:** Descriptive data of intention to leave at T1, T2 and T3; mean values (*SD*) of demands and resources at T1. Comparisons of mean values between assistant nurses and RNs

	Time Group differences^a^	Assistant nurses *M* (*SD*)	RNs *M* (*SD*)
**Intention to leave**	T1	2.49 (1.03)	2.56 (0.98)
	T2	2.52 (1.03)	2.58 (1.03)
	T3***	2.45 (0.99)	2.66 (0.98)
**DEMANDS**			
Quantitative demands	T1***	2.65 (0.66)	2.94. (0.66)
Emotional demands	T1	3.51 (0.68)	3.49 (0.57)
Work pace	T1***	3.48 (0.64)	3.62 (0.59)
Role conflict	T1***	2.60 (0.75)	2.75 (0.72)
Illegitimate tasks	T1	2.75 (0.89)	2.81 (0.82)
**RESOURCES**			
Workflow	T1*	3.58 (0.64)	3.48 (0.72)
Influence at work	T1	3.01 (0.67)	3.06 (0.67)
Possibilities for development	T1***	3.86 (0.66)	4.05 (0.64)
Predictability	T1***	3.45 (0.75)	3.29 (0.67)
Social support from colleagues	T1	4.12 (0.60)	4.13 (0.50)
Social support from supervisor	T1	3.24 (0.90)	3.26 (0.86)
Recognition	T1	3.59 (0.73)	3.62 (0.68)
Quality of leadership	T1	3.31 (0.83)	3.27 (0.80)
Vertical trust	T1	3.22 (0.84)	3.27 (0.84)
Organizational justice	T1	3.22 (0.60)	3.19 (0.61)
Humanity	T1	3.92 (0.73)	3.96 (0.63)
Organizational clarity	T1	4.38 (0.56)	4.26 (0.45)
Interprofessional collaboration	T1*	2.83 (0.56)	2.76 (0.55)

^a^ Differences in mean values between assistant nurses’ and RNs’.

*** *p *< .001;

* *p *< .05.

### 3.2 Associations between job demands, job resources and intention to leave

The results of the correlation analysis showed that job demands at T1 in most of the analyses predicted higher levels of assistant nurses' intentions to leave at both T2 and T3 (see Table 5). Furthermore, all job demands predicted RNs' intentions to leave at T2. Quantitative demands, role conflict and illegitimate tasks also predicted RNs' intentions to leave at T3. In general, there were stronger correlations between job demands and intention to leave among assistant nurses compared with RNs (see Table 5).

**Table 5:** Associations between resources and demands at T1 and intention to leave at T2 and T3 among assistant nurses and RNs

	Assistant nurses Intention to leave T2	Assistant nurses Intention to leave T3	RNs Intention to leave T2	RNs Intention to leave T3
Demands T1				
Quantitative demands	0.38 (0.14)***	0.36 (0.12)***	0.20 (0.04)***	0.21 (0.04)***
Emotional demands	0.36 (0.13)***	0.30 (0.08)***	0.14 (0.02)*	0.07 (0)
Work pace	0.37 (0.13)***	0.29 (0.08)***	0.16 (0.02)**	0.12 (0.01)
Role conflict	0.25 (0.06)***	0.14 (0.01)	0.23 (0.05)***	0.20 (0.03)**
Illegitimate tasks	0.34 (0.11)***	0.14 (0.01)	0.20 (0.04)***	0.24 (0.05)***
Resources T1				
Workflow	−0.25 (0.05)***	−0.12 (0.02)	−0.28 (0.08)***	−0.24 (0.05)***
Influence at work	−0.13 (0.02)	−0.18 (0.02)*	−0.26 (0.07)***	−0.28 (0.08)***
Possibilities for development	−0.08 (0)	−0.07 (0)	−0.13 (0.02)^ǂ^	−0.16 (0.02)*
Predictability	−0.22 (0.04)**	−0.22 (0.03)^ǂ^	−0.31 (0.09)***	−0.33 (0.11)***
Social support from colleagues	−0.12 (0.01)	−0.16 (0.02)	−0.13 (0.01)*	−0.09 (0)
Social support from supervisor	−0.01 (0)	−0.12 (0.01)	−0.23 (0.05)***	−0.18 (0.03)**
Recognition	−0.22 (0.04)**	−0.29 (0.08)***	−0.31 (0.09)***	−0.31 (0.09)***
Quality of leadership	−0.17 (0.02)*	−0.22 (0.04)*	−0.26 (0.06)*	−0.22 (0.04)**
Vertical trust	−0.23 (0.05)***	−0.22 (0.04)***	−0.25 (0.06)***	−0.33 (0.11)***
Organizational justice	−0.14 (0.04)	−0.21 (0.04)*	−0.27 (0.07)***	−0.25 (0.06)***
Humanity	−0.06 (0)	−0.15 (0.01)***	−0.27 (0.07)***	−0.23 (0.05)***
Organizational clarity	−0.15 (0.02)^ǂ^	−0.28 (0.07)***	−0.12 (0.01)^ǂ^	−0.22 (0.04)***
Interprofessional Collaboration	−0.26 (0.08)***	−0.17 (0.02)	−0.28 (0.08)***	−0.27 (0.07)***

Note

Unstandardized b‐coefficients, (Δ*R* ^2^).

^ǂ^ *p *< 0.10.

* *p *< .05.

** *p *< .01.

*** *p *< .001.

Lower levels of job resources in approximately half of the analyses predicted higher levels of intention to leave at both T2 and T3 among assistant nurses (Table 5). Almost all investigated resources predicted RNs' intentions to leave at both T2 and T3.

### 3.3 Moderating effects of job resources

In the last step of the analysis, specific resources' associations with specific demands and intentions to leave at T1, T2 and/or T3 were calculated (the results are summarized in Table 6). None of the resources affected associations between quantitative demands or illegitimate tasks and intention to leave at T2 or T3 in the stratified analysis of assistant nurses and RNs. Likewise, but only for assistant nurses' intentions to leave at T2 or T3, none of the resources affected the associations between work demands, role conflict and illegitimate tasks. For assistant nurses, organizational clarity and interprofessional collaboration affected the association between emotional demands and intention to leave such that intention to leave decreased at T2 and T3, respectively.

**Table 6:** Intention to leave regressed on job demands and resources

	Assistant nurses Intention to leave T2	Assistant nurses Intention to leave T3	RNs Intention to leave T2	RNs Intention to leave T3
Emotional demands				
Emotional demands	–	–	0.26*	–
Workflow	–	–	−0.37***	–
Emotional demands × Workflow	–	–	0.20*	–
Emotional demands	0.58***	–	–	–
Organizational clarity	−0.35*	–	–	–
Emotional demands × Organizational clarity	0.36*	–	–	–
Emotional demands	–	0.48***	–	–
Interprofessional collaboration	–	−0.42*	–	–
Emotional demands × Collaboration	–	0.55*	–	–
Work pace				
Work pace	–	–	0.28*	0.25*
Social support from colleagues	–	–	−0.41**	−0.42**
Work pace × Social support from colleagues	–	–	0.48*	0.68**
Work pace	–	–	–	0.24*
Social support from supervisor	–	–	–	−0.25**
Work pace × Social support from supervisor	–	–	–	0.31*
Work pace	–	–	–	0.27*
Vertical trust	–	–	–	−0.43***
Work pace × Vertical trust	–	–	–	0.31*
Work pace	–	–	–	0.40*
Humanity	–	–	–	−0.37***
Work pace × Humanity	–	–	–	0.29***

Note

Only the results of models showing that a resource significantly affected the association between demands and intentions to leave are presented in the table. Unstandardized b‐coefficients.

* *p *< .05.

** *p *< .01, *p *< .01.

*** *p *< .001.

For RNs, the following resources affected the associations between demands and intention to leave. Workflow was associated with emotional demands (T2); social support from colleagues was associated with work pace (T2 and T3); social support from manager, vertical trust and humanity were associated with work pace (T3).

**Figure 2:**
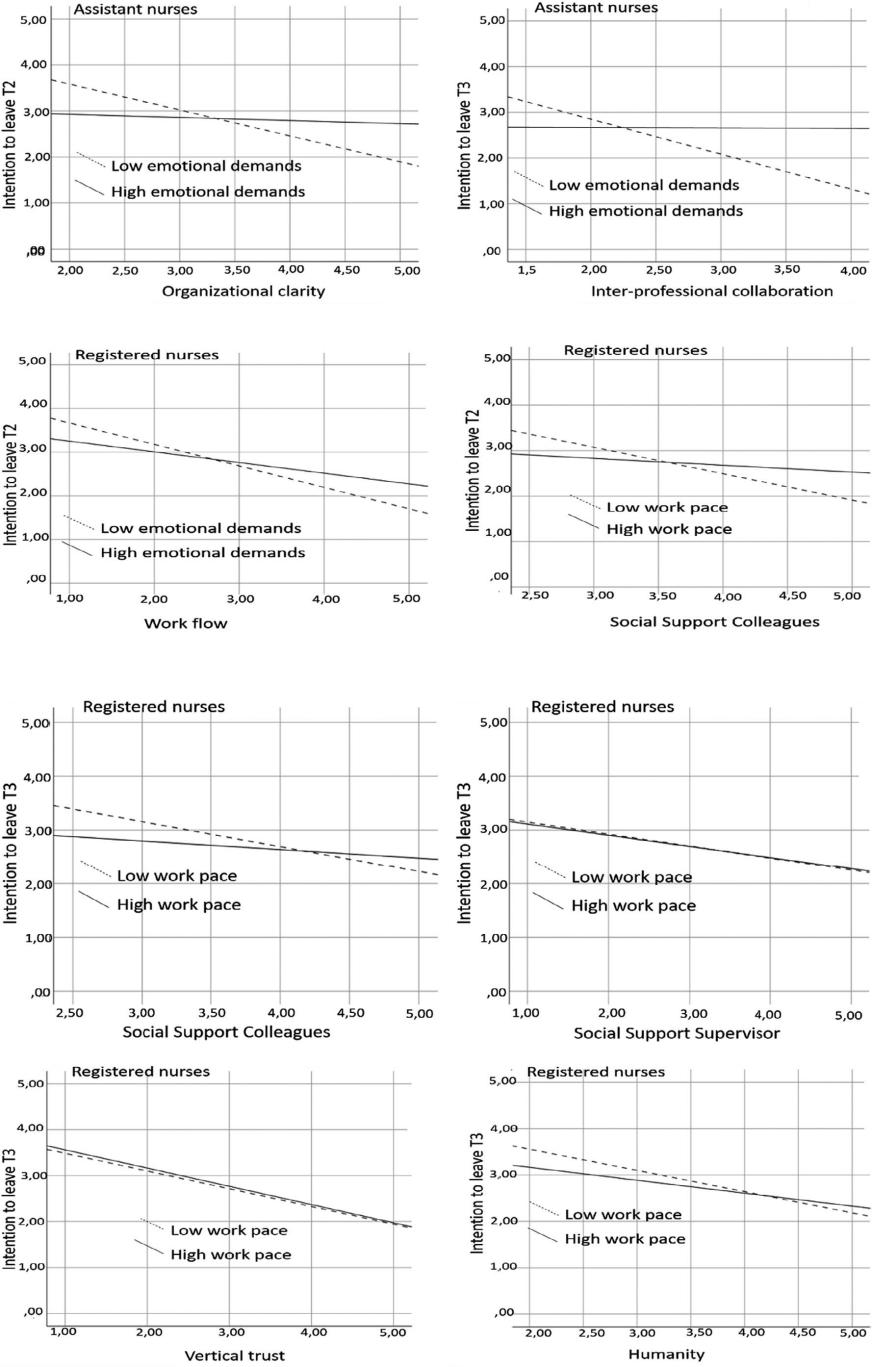
The associations between low versus high demands and intention to leave under different levels of the job resources

The associations between low, respectively, high demands with low, respectively, high demands with intention to leave under different levels of moderating job resources are presented in Figure 2. From an examination of these moderating effects, it can be concluded that, in general, higher emotional demands in combination with higher degrees of support, no matter what kind of support, are typically linked to lower degrees of intentions to leave.

## 4 DISCUSSION

This study investigated the kinds of job demands and resources that predicted intention to leave among assistant nurses and RNs and whether specific resources had moderating effects on the associations between specific demands and their intention to leave. The result showed that most of the investigated demands predicted intention to leave among both assistant nurses and RNs. Furthermore, most resources predicted RNs' intentions to leave, while for assistant nurses, approximately half of the investigated resources predicted their intentions to leave. The wide variety of job demands and resources that predict intentions to leave are in line with previous studies (Chan et al., 2013; Choi et al., 2013; Hasselhorn et al., 2005; Hayes et al., 2012).

For assistant nurses, only organizational clarity and interprofessional collaboration were found to moderate the negative association between emotional demands and assistant nurses' intentions to leave. For RNs, workflow was a functional resource that moderated the negative association between emotional demands and their intentions to leave. Likewise, social support from colleagues and supervisors and vertical trust moderated the negative association between high work pace and intention to leave.

It is interesting to note that none of the investigated resources moderated the association between quantitative demands or illegitimate tasks and any of the studied occupational groups' intentions to leave. These results can be compared with a recent study with employees from different kinds of organizations, including public care organizations, pointing out that resources such as decision latitude and social capital did not moderate the association between job demands and increased risk on burnout (Ståhl et al., 2018). The results confirm Moloney et al. (2018) conclusion on the importance of measures contributing to manageable workload for reducing intentions to leave.

Job demands were in general more strongly correlated with assistant nurses' intentions to leave, although they rated lower demands compared with RNs. Similarly, assistant nurses rated resources higher and it was from this perspective a surprising finding that almost none of the resources moderated the association between demands and assistant nurses' intentions to leave. One explanation for this might be that RNs' educational background and possibilities for specialization make them better prepared for handling high demands in work. The fact that assistant nurses are more negatively affected by demands could also possibly be explained by socio‐economic factors (i.e. assistant nurses have lower income and lower job status compared with RNs). In general, having a job with lower social status includes a greater exposure to both physical and psychosocial risks. Also, lower income is correlated with poorer health conditions (Adler & Newman, 2002). One previous study reported that licensed practical nurses more frequently reported that physical demands, inability to provide safe competent care, too much responsibility, health problems, burnout, more time with family and poor salary contributed to their intentions to leave compared with RNs (Havaei et al., 2016). It would be of interest to investigate further whether job satisfaction and health status moderate the negative impact of job demands on assistant nurses' intentions to leave. Another explanation for differences between assistant nurses and RNs could possibly be found in labour market conditions. RNs have more options for further training and specializing, opening up for more opportunities in the job market. This might mean that assistant nurses to a greater extent are locked into a job they might want to leave. Among the investigated predictors, quantitative demands tended to have the strongest correlation with assistant nurses' intentions to leave. Another study showed increased quantitative demands over time for both RNs and assistant nurses, especially for assistant nurses, which might be an explanation for the stronger effect of quantitative demands on assistant nurses' intentions to leave over time (Dellve et al., 2016).

Several of the resources that moderated the relationship between demands and RNs' intentions to leave in our study could be related to social capital at the workplace (Kouvonen et al., 2006) including social support from employees and managers, vertical trust and humanity. Social capital was previously shown in a cross‐sectional study to be associated with healthcare professionals' intentions to leave. However, that study did not find that social capital moderated the relationship between demands and intentions to leave (Strömgren, 2017b). The differences in results might be explained by the fact that Strömgren's, (2017b) study did not make stratified analyses of different occupational groups (i.e. physicians, RNs and assistant nurses) and that the study included the sum of both quantitative demands and work pace.

Previous research suggests a “triple‐match‐principle,” that is that interaction effects occur if demands, resources and outcomes match. This means that buffering effects most probably occur when the qualitative aspects of job demands, job resources and outcomes match (De Jonge & Dormann, 2006). Intention to leave can, in this context, be considered a motivational outcome which implies that the most likely finding is that interactions between job demands and resources represent motivational factors. Therefore, one explanation could be that a supportive and trusting work culture contributes to the feeling that a high work pace is more manageable and thus might also be experienced as a motivating demand. In the same vein, one might argue that high emotional demands connected to, for example, patient work are easier to deal with and might even be a motivational factor if the overall work is well organized and is not adding to the emotional strain. This could explain why good workflow, organizational clarity and good interprofessional collaboration are moderating factors for emotional strain.

More research is needed on how to develop a supportive work culture and an organization of work that contribute to reducing the negative impact of excessive job demands. Work breaks have in this context been suggested as an organizational resource that interact with perceptions of social job resources and job demands (Wendsche et al., 2017) and, for example, also moderates the negative effects that understaffing usually have on staff turnover (Wendsche et al., 2017). Explanations for this might be that breaks give recovery opportunities including opportunities to develop a positive and supportive social climate. More applied research is however needed on how organizational measures, such as scheduled work breaks, could contribute to structuring a manageable workday and foster a supportive and trusting work culture.

In line with previous research, the present results imply the importance of healthcare organizations to set aside time for development work aiming at improving resource‐giving work conditions at shop floor level (Dellve et al., 2015). Our results suggest that development work aiming at improvements of workflow, interprofessional collaboration and relationships between managers and employees might be important for decreased intentions to leave. It can also be considered specifically important to develop functional resources supporting younger nurses as previous research show that they have higher degrees of intentions to leave (Flinkman et al., 2010). The results specifically point to the importance of developing social capital as an organizational resource for staff retention in healthcare organizations, which is in line with other research showing the importance of social capital for healthcare professionals' engagement and job satisfaction (Strömgren, 2017a).

### 4.1 Limitations

One limitation of this study is that the participants were not asked to rate the degree to which demands and resources contributed to their intentions to leave. Another limitation is that eventual re‐organizations, change in management or change in policies that might be associated with levels of intention to leave were not included. Qualitative complementary studies are recommended to explain why there are differences in experiences of job demands and resources and their relationship with intention to leave among the two studied occupational groups. In addition to organizational and work‐related conditions, explanations for the results might also be found in labour market conditions and retirement benefit systems. The study includes extensive statistical tests, and therefore, the risk that some of the results could be false positive must be acknowledged. A weakness of the study is thus that *p*‐values have not been adjusted for multiple tests. One implication of this is that the results with *p*‐values above .01 should be interpreted with caution. More studies are needed to confirm the differences between assistant and RNs and also the results on the moderating effects of resources.

## 5 CONCLUSIONS

The results of this study indicate that job demands better predict assistant nurses' intentions to leave, whereas resources better predict RNs' intentions to leave. These results contribute to mapping out how functional resources and specific demands interact and are associated with nurses' intentions to leave. The study reveals that for RNs, there are several specific resources that can counteract the negative association between emotional demands respectively work pace and intention to leave. It should be a priority for future practice and research to examine how specific resources can be developed to counteract the negative impact of high demands on assistant nurses' intentions to leave. The results imply that nurse managers need to consider how functional organizational resources can be developed specifically for assistant nurses to prevent the negative impact of demands on their intentions to leave. Finally, the results show the importance of directly lowering job demands for developing sustainable work conditions and promoting staff retention among both assistant nurses and RNs.

## CONFLICT OF INTEREST

None of the authors have any conflict of interest to state.

## AUTHOR CONTRIBUTIONS

AE, GJ and LD: Substantial contributions to conception and design, analysis and interpretation of data. AE and LD: Acquisition of data. AE, GJ and LD: Drafting the manuscript, revising it critically for important intellectual content and gave final approval of the version to be published. Thus, all authors have participated sufficiently in the work to take public responsibility for appropriate portions of the content; agreed to be accountable for all aspects of the work in ensuring that questions related to the accuracy or integrity of any part of the work are appropriately investigated and resolved.

## ETHICAL APPROVAL

The study was approved by the regional ethical research committee in Stockholm, Sweden (ref: 2012/94‐31/5 and ref: 2014/1993‐31/5).

## Data Availability

The data that support the findings of this study are available on request from the corresponding author, AE. The data are not publicly available due to their containing information that could compromise the privacy of research participants.
